# Two livebirths achieved in cases of hypergonadotropic hypogonadism nonobstructive azoospermia, treated with GnRH agonist and gonadotrophins: a case series and review of the literature

**DOI:** 10.5935/1518-0557.20240039

**Published:** 2024

**Authors:** Mauro Bibancos de Rose, Arhon Bizelli Sicard, Natalia Alvarenga Aguiar, Beatriz de Oliveira Onório, Antonio Alberto Rodrigues Almendra, Wagner Eduardo Matheus, Andrea Garolla, Carlo Foresta, Daniela Paes de Almeida Ferreira Braga, Amanda Souza Setti, Edson Borges Jr.

**Affiliations:** 1 Fertility Medical Group. São Paulo - SP, Brazil; 2 Urology Department - Pontifícia Universidade Católica de Campinas - PUCAMP. Campinas - SP, Brazil; 3 Urology Department - Universidade Estadual de Campinas - UNICAMP. Campinas - SP, Brazil; 4 Unit of Andrology and Reproductive Medicine, Department of Medicine - University of Pandova. Pandova - PD, Italy; 5 Fertility/FERTGROUP-Medicina Reprodutiva. São Paulo - SP, Brazil

**Keywords:** infertility, azoospermia, assisted reproduction, GnRH agonist, intracytoplasmic sperm injection

## Abstract

Non-obstructive azoospermia (NOA) is the most severe form of male factor infertility. It results form from either primary or secondary testicular failure. Here, we report cases of two patients with NOA due to maturation arrest and increased serum FSH, treated with GnRH agonist and gonadotrophins. The two NOA patients underwent a pharmacological treatment consisting of pituitary desensibilization using a GnRH agonist and testicular stimulation using menotropin. Testicular stimulation started one month after the beginning of GnRH agonist treatment. The female partner underwent controlled ovarian stimulation (COS) followed by intracytoplasmic sperm injection (ICSI). On the third day of the cycle, menotropin daily doses was administered. When at least one follicle ≥14 mm was visualized, pituitary blockage was performed using GnRH antagonist ganirelix. When three or more follicles attained a mean diameter of ≥17 mm, triptorelin acetate was administered to trigger final follicular maturation. Oocyte retrieval was performed 35 hours later. After treatment, male partner blood levels of the FSH, LH, decreased and total testosterone were increased. Spermatozoa was observed after semen collection in both cases. After COS, oocytes were retrieved and ICSI was performed. Embryos were biopsied for preimplantation genetic testing (PGT) and those considered euploidy were transferred resulting in positive implantation, ongoing pregnancy, and livebirth on both cases. In this report we present a successful strategy for hypergonadotropic hypogonadism AOA men, as an alternative approach to the surgical testicular sperm recovery. Nevertheless, prospective randomized trials are needed to confirm our findings.

## INTRODUCTION

Azoospermia, the absence of spermatozoa in the ejaculate is the most severe form of male factor infertility and it is classified as obstructive azoospermia (OA) or non-obstructive azoospermia (NOA), each having very different etiologies (Wosnitzer *et al.,* 2014). Non-obstructive azoospermia occurs when there is an impairment to spermatogenesis and it results from either primary testicular failure (related to an intrinsic defect in the testicles) or secondary testicular failure (usually due to hypothalamic-pituitary disorders), leading to a hypergonadotropic hypogonadism or hypogonadotropic hypogonadism, respectively (Sengupta *et al.,* 2021; Tharakan *et al.,* 2021). According with its histological findings NOA is classified as hypospermatogenesis, maturation arrest, and Sertoli-cell only (SCO) (McLachlan *et al.,* 2007).

Azoospermic patients were considered sterile but, with the advent of testicular sperm extraction (TESE) and micro TESE (Schoysman *et al.,* 1993) and assisted reproductive technology (ART), men with azoospermia have a chance to father their genetically own child with intracytoplasmic sperm injection (ICSI). Although TESE and micro TESE followed by ICSI may be possible in NOA patients, it is and invasive and expensive technic, and there is a possibility that sperm cannot be recovered by this technique (Deruyver *et al.,* 2014; Bernie *et al.,* 2015).

Alternatively, NOA patients would benefit from hormonal treatment (Shiraishi *et al.,* 2012). Indeed, male fertility depends on a complex crosstalk of hormones (Singh *et al.,* 2019). Gonadotropin-releasing hormone (GnRH) synthesized by the hypothalamus promotes the secretion of gonadotropins by the anterior pituitary. FSH acts on the Sertoli cells, promoting spermatogonial maturation, and in Leydig cells, LH promotes the synthesis and release of testosterone (Dutta *et al.,* 2019; Singh *et al.,* 2019).

In male presenting with hypogonadotropic hypogonadism, restoration of FSH action may improve sperm parameters and, therefore, fertility (Rastrelli *et al.,* 2014). In these cases, fertility can be restored using either GnRH or exogenous gonadotropins (Casarini *et al.,* 2020). Meta-analyses of the published literature suggest that FSH treatment to infertile men, both spontaneously and during assisted reproduction, improves pregnancy rates (Santi *et al.,* 2015; Behre, 2019).

However, in hypergonadotropic hypogonadism, gonadotropin levels are raised due to a lack of the negative feedback from testicular testosterone, estradiol, or inhibin B, because of an insufficient or nonexistent function of the testicles (Sengupta *et al.,* 2021). Eventually, the FSH level becomes so high that down-regulation of FSH receptors occurs. As demonstrated by in vivo and in vitro ancient studies, desensitization and downregulation of FSH signaling in Sertoli cells may be induced by the chronic stimulation of FSH (Gnanaprakasam *et al.,* 1979; Santi *et al.,* 2015).

For the present study, we report cases of two patients with NOA due to maturation arrest and increased serum FSH, treated with GnRH agonist and gonadotrophins. After the treatment the spermatogenesis was restored, and successful pregnancies and livebirth were achieved.

## CASE DESCRIPTION

### Case 1

A 40-year-old man underwent diagnostic examinations to detect the possible causes of infertility. The patient was trying to conceive for over one year with a healthy female partner.

At the first appointment in November 2019, hormone profile was requested, and physical examination was performed. The blood levels of the FSH, LH, and total testosterone were 14.2 mIU/mL, 2.2 mIU/mL, and 290.4 ng/dL, respectively. Physical examination revealed testis size of 13 mL bilateral, normal consistency, presence of the vasa deferentia with normal consistency, epididymis with normal consistency, and absence of varicoceles. Semen sample was collected in the laboratory by masturbation and after liquefaction for 30 minutes the patient was diagnosed with azoospermia. Diagnostic histological testicular biopsy was performed and early-stage maturation arrest at the spermatogonia stage was observed.

His partner was 32-year-old with regular menstrual cycle and no dysmenorrhea. Pelvic ultrasound revealed a normal uterus, both ovaries, normal antral follicle count, and serum level of A mullerian hormone (AMH): 1.81 mg/mL.

The patient underwent a pharmacological treatment consisting of pituitary desensibilization using a GnRH agonist (leuprorelin acetate 3.75 mg, Lupron Depot®; AbbVie Inc., North Chicago, Illinois, USA) for 4 months and testicular stimulation using menotropin 1,200 IU (Menopur^®^, Ferring Pharmaceuticals, Saint-Prex, Switzerland), every other day, and hCG 5,000 IU (Choriomon®, Meizler, UCB, Biopharma, Belgium), every two weeks, both for three months. Testicular stimulation started one month after the beginning of GnRH agonist treatment

Post treatment blood levels of the FSH, LH, and total testosterone were 3.01 mIU/mL, 0.2 mIU/mL and 100.1 ng/dL, respectively. Semen sample was collected again, and sperm count was 0.3 M/mL, total motility: 74%, and progressive motility: 63%.

The female partner underwent controlled ovarian stimulation (COS) followed by ICSI. On the third day of the cycle, menotropin (Menopur®, Ferring Pharmaceuticals, Saint-Prex, Switzerland) daily doses was administered. When at least one follicle ≥14 mm was visualized, pituitary blockage was performed using GnRH antagonist ganirelix (Orgalutran®, Organon, Oss, Netherlands). When three or more follicles attained a mean diameter of ≥ 17 mm, triptorelin acetate (Gonapeptyl®, Ferring Pharmaceuticals, Saint-Prex, Switzerland) was administered to trigger final follicular maturation. Oocyte retrieval was performed 35 hours later.

On November 2021, 20 follicles were aspirated and 16 oocytes and 12 matures oocytes were retrieved, respectively. Sperm sample was prepared using a two-layered density gradient centrifugation technique (50% and 90% Isolate, Irvine Scientific, Santa Ana, CA, USA). Intracytoplasmic sperm injection was performed in all mature oocytes and 6 embryos were obtained, in which 4 achieved the blastocyst stage. Two embryos were biopsied for preimplantation genetic testing (PGT) and both were considered euploidy. Two euploid embryos were transferred and one implanted, resulting in a successful pregnancy and livebirth.

### Case 2

A 36-year-old man trying to conceive for over one year with a healthy female partner, had had history of primary infertility and underwent previous ART cycles with donor semen. At the first appointment in September 2020, hormone profile was requested, and physical examination was performed. The blood levels of the FSH, LH, and total testosterone were 40.19 mIU/mL, 32.41 mIU/mL and 270.40 ng/dL, respectively. Physical examination revealed testis size of 12 mL, bilateral, normal consistency, presence of the vasa deferentia with normal consistency, epididymis with normal consistency, and absence of varicoceles. Semen sample was collected in the laboratory by masturbation and after liquefaction for 30 minutes the patient was diagnosed with azoospermia. Diagnostic histological testicular biopsy was performed and early-stage maturation arrest at the spermatocyte II stage was observed.

His partner was 34-year-old with regular menstrual cycle and no dysmenorrhea. Pelvic ultrasound revealed a normal uterus, both ovaries, normal antral follicle count, and the blood levels of AMH: 1.62 mg/mL.

The patient underwent a pharmacological treatment consisting of pituitary desensibilization using a GnRH agonist (leuprorelin acetate 3.75 mg, Lupron Depot®; AbbVie Inc., North Chicago, Illinois, USA) for 4 months and testicular stimulation using menotropin 1,200 IU (Menopur®, Ferring Pharmaceuticals, Saint-Prex, Switzerland), every other day, and hCG 5,000 IU (Choriomon®, Meizler, UCB, Biopharma, Belgium), every two weeks, both for three months. Testicular stimulation started one month after the beginning of GnRH agonist treatment.

Post treatment blood levels of the FSH, LH, and total testosterone were 16.11 mIU/mL, 0.36 mIU/mL and 345.12 ng/dL, respectively. Semen sample was collected again, and sperm count was 0.01 M/mL, total motility: 99%, and progressive motility: 75%.

The female partner underwent controlled ovarian stimulation (COS) followed by ICSI.

On the third day of the cycle, menotropin (Menopur®, Ferring Pharmaceuticals, Saint-Prex, Switzerland) daily doses was administered. When at least one follicle ≥14 mm was visualized, pituitary blockage was performed using gonadotropin-releasing hormone (GnRH) antagonist ganirelix (Orgalutran^®^, Organon, Oss, Netherlands). When three or more follicles attained a mean diameter of ≥ 17mm, triptorelin acetate (Gonapeptyl®, Ferring Pharmaceuticals, Saint-Prex, Switzerland) was administered to trigger final follicular maturation. Oocyte retrieval was performed 35 hours later.

On April 2021, 24 follicles were aspirated and 15 oocytes and 11 matures oocytes were retrieved respectively. Sperm sample was prepared using a two-layered density gradient centrifugation technique (50% and 90% Isolate, Irvine Scientific, Santa Ana, CA, USA). Intracytoplasmic sperm injection was performed in all mature oocytes and 6 embryos were obtained, in which 4 achieved the blastocyst stage. Embryos were biopsied for PGT and 3 were euploidy. Two embryos were transferred and one implanted resulting in a successful pregnancy and livebirth.

## DISCUSSION

Historically azoospermic men were considered infertile. However, ICSI has revolutionized the treatment of male infertility, enabling fertilization of oocytes using a single spermatozoon (Palermo *et al.,* 1992). By using ICSI, since 1995 biological fatherhood has been possible for men with NOA though surgical retrieval of testicular spermatozoa (Schoysman *et al.,* 1993).

Nevertheless, sperm recovery is not a trivial procedure in NOA men, since the spermatogenesis in these cases is only present in small areas, if any [4] and usually multiple procedures are necessary, which may result in significant testicular parenchyma loss and in blood supply impairment, eventually resulting in testicular atrophy (Schlegel & Su, 1997)England.

Indeed, testicular surgical procedure has been suggested a procedure that may result in complications, such as d haematoma, devascularization, inflammation, ultimately leading to scars and calcification (reviewed in Eliveld *et al.,* 2018). Furthermore, a decrease in serum testosterone levels after a TESE procedure has been describe (Donoso *et al.,* 2007; Shin & Turek, 2013), which can subsequently lead to hypogonadism.

In previous published meta-analysis, a transient but significantly decreased total testosterone levels after TESE, that recover to baseline levels after 18-26 months was observed. The available information on signs and symptoms of hypogonadism after TESE suggests that some men may experience erectile dysfunction, together with depression and anxiety, and a decrease in testicular size (Eliveld *et al.,* 2018).

Higher serum FSH levels and smaller testicular volumes are associated with more severe testicular histopathology in men with NOA (Gudeloglu & Parekattil, 2013). The pharmacological treatment for male infertility, mainly through the administration of gonadotropins, has been carried out for over 50 years (Alexandre, 1978; Chehval & Mehan, 1979), and is still the treatment of choice in male infertility due to hypogonadotropic hypogonadism (Duca *et al.,* 2019).

FSH has been proved to improve sperm parameters and pregnancy rates in normogonadotropic oligozoospermic patients (Casamonti *et al.,* 2017; Garolla *et al.,* 2017), in patients with sperm maturation arrest (Barbotin *et al.,* 2017), and those undergoing TESE (Cocci *et al.,* 2018). However, patients with high serum FSH may not benefit from this therapy. Excess gonadotropin exposure has the potential for desensitizing Leydig and Sertoli cells and may decrease responsiveness to LH and FSH. Hypothalamic-pituitary-gonadal axis suppression followed reactivation is an artificial way to re-sensitize Leydig and Sertoli cells resulting in improved spermatogenesis (Foresta *et al.,* 2009).

Considering this Foresta *et al.* (2004), were able to improve the Sertoli cell function by reduction high FSH plasma concentrations by administration of a GnRH agonist. In a subsequent controlled, randomized trial, they analyzed the effect of FSH treatment in patients with oligozoospermia after desensitization of FSH receptors and observed a significant improvement of sperm parameters and a reduction of sperm aneuploidies in patients with high basal FSH plasma concentrations (Foresta *et al.,* 2009).

In 2012, Shiraishi *et al.* (2012), tried to reduce the gonadotropin secretion by pituitary blockage using high-dose hCG. As a result, the gonadotropins levels in NOA patients were decreased and the testicular spermatogenesis was improved, indicating that pituitary blockage may be an efficient treatment for NOA patients.

More recently, high level of endogenous gonadotropins was suppressed using a GnRH agonist and testis was stimulated by hMG and hCG, resulting in improved spermato-genesis in patients diagnosed with hypospermatogenesis. However, in this trial in only 2 out of 25 patients sperm was retrieved (Hu *et al.,* 2018).

Based on Foresta studies (Foresta *et al.,* 2004; 2009), we hypothesized that if Sertoli cell function and sperm parameters were improved by FSH receptors desensitization in oligospermic patients, this treatment would do good for NOA men. Indeed, while some males are thought to have lifelong NOA, other, who are initially oligospermic, may lose the ability to produce sperm in the ejaculate reliably over time. Although it remains unclear why some men progress to overt NOA, mechanisms for both conditions may be the similar (Song *et al.,* 2010; Patel *et al.,* 2023). In the present study, two NOA men with maturation arrest and high serum FSH were treated with GnRH agonist, aiming to suppress the hypothalamic-pituitary-gonadal axis. Following pituitary blockage, patients were stimulated with menotropin and hCG. After hormonal treatment, both presented normal FSH levels and spermatozoa was observed on the ejaculates. The female partner underwent controlled ovarian stimulation followed by ICSI. On both cases two embryos were transferred, resulting in successful single pregnancy and livebirth.

Treatment with FSH has been introduced to increase sperm concentration, spermatogonial population, or pregnancy rates in normogonadotropic oligozoospermic (Garolla *et al.,* 2017) and azoospermic subjects (Barbotin *et al.,* 2017). For men with infertility and low gonadotropin concentrations presenting an authentic hypogonadism, therapy with gonadotropins can result in significant increases in sperm output over time. Improvements in sperm output are notable after 3 to 6 months of therapy, which can lead to conception in many cases (Liu *et al.,* 2009; Young *et al.,* 2019).

Men presenting with hypergonadotropic hypogonadism are treated with medical therapies alone or in combination with ART. Selective estrogen receptor modulators, aromatase inhibitors, gonadotropins, and their combinations are among the available treatment options (Kalkanli *et al.,* 2021).

For the present study, instead of using an FSH-only gonadotropin preparation, menotropin, a mixture of FSH and LH, was used, associated with hCG. Indeed, both testosterone synthesis and male fertility result from the delicate coordination throughout pituitary-gonadal axis. While LH triggers the Leydig cells to produce testosterone, FSH triggers and sustains the spermatogenesis within the exocrine part of the testes (Salonia *et al.,* 2019). Therefore, a preparation with FSH and LH function would a more “physiological” stimulation.

One could ask why in the present study the patients was submitted to such a long treatment instead of trying a surgical testicular sperm retrieval procedure, which would be a successful and much shorter treatment. The choice for the hormonal treatment was due to the fact that female partners were young and fertile, therefore a long waiting time would not compromise the ovarian reserve, nor the oocyte quality. Additionally surgical testicular sperm retrieval is an invasive procedure not free of adverse results, and finally, even though ICSI with testicular sperm have achieved good results, mainly due to advances in sperm preparation and selection methods, recent studies using time-lapse imaging (TLI) technology demonstrated that spermatozoa that have completed maturation, during their transit through the male reproductive tract, generates embryos with faster cell divisions, more rapid compaction (Karavani *et al.,* 2021), faster second cell cycle, faster blastulation (Lammers *et al.,* 2015), and higher implantation rates (Karavani *et al.,* 2021).

On conclusion, this report presents a successful strategy for hypergonadotropic hypogonadism AOA men, as an alternative approach to the surgical testicular sperm recovery. Nevertheless, a prospective randomized trial is needed to confirm our findings.
