# Corrigendum to “Two livebirths achieved in cases of hypergonadotropic hypogonadism nonobstructive azoospermia, treated with GnRH agonist and gonadotrophins: a case series and review of the literature” [JBRA Assist Reprod. 2024;28(3):521-525]

**DOI:** 10.5935/1518-0557.20250001

**Published:** 2025

**Authors:** Mauro Bibancos de Rose, Arhon Bizelli Sicard, Natalia Alvarenga Aguiar, Beatriz de Oliveira Onório, Antonio Alberto Rodrigues Almendra, Wagner Eduardo Matheus, Andrea Garolla, Carlo Foresta, Daniela Paes de Almeida Ferreira Braga, Amanda Souza Setti, Edson Borges Jr.

**Affiliations:** 1 Fertility Medical Group. São Paulo - SP, Brazil; 2 Urology Department - Pontifícia Universidade Católica de Campinas - PUCAMP. Campinas - SP, Brazil; 3 Urology Department - Universidade Estadual de Campinas - UNICAMP. Campinas - SP, Brazil; 4 Unit of Andrology and Reproductive Medicine, Department of Medicine - University of Pandova. Pandova - PD, Italy; 5 Fertility/FERTGROUP-Medicina Reprodutiva. São Paulo - SP, Brazil

The authors regret that in page 522: CASE DESCRIPTION Case 1, it was described that: …” The patient underwent a pharmacological treatment consisting of pituitary desensibilization using a GnRH agonist (leuprorelin acetate 3.75 mg, Lupron Depot^®^; AbbVie Inc., North Chicago, Illinois, USA) for 4 months and testicular stimulation using menotropin 1200 IU (Menopur^®^, Ferring Pharmaceuticals, Saint-Prex, Switzerland), every other day, and hCG 5,000 IU (Choriomon^®^, Meizler, UCB, Biopharma, Belgium), every two weeks, both for three months”

The dose of menotropin used for testicular stimulation was incorrectly reported. The correct dose should be read as testicular stimulation using menotropin 1200 IU. Therefore, the correct text should be: “The patient underwent a pharmacological treatment consisting of pituitary desensibilization using a GnRH agonist (leuprorelin acetate 3.75 mg, Lupron Depot^®^; AbbVie Inc., North Chicago, Illinois, USA) for 4 months and testicular stimulation using menotropin 150 IU (Menopur^®^, Ferring Pharmaceuticals, Saint-Prex, Switzerland), every other day, and hCG 5,000 IU (Choriomon^®^, Meizler, UCB, Biopharma, Belgium), every two weeks, both for three months”.

The same mistake was made for Case Report 2. Where it reads: “The patient underwent a pharmacological treatment consisting of pituitary desensibilization using a GnRH agonist (leuprorelin acetate 3.75 mg, Lupron Depot^®^; AbbVie Inc., North Chicago, Illinois, USA) for 4 months and testicular stimulation using menotropin 1200 IU (Menopur^®^, Ferring Pharmaceuticals, Saint-Prex, Switzerland), every other day, and hCG 5,000 IU (Choriomon^®^, Meizler, UCB, Biopharma, Belgium), every two weeks, both for three months.” it should read: “The patient underwent a pharmacological treatment consisting of pituitary desensibilization using a GnRH agonist (leuprorelin acetate 3.75mg, Lupron Depot^®^; AbbVie Inc., North Chicago, Illinois, USA) for 4 months and testicular stimulation using menotropin 150IU (Menopur^®^, Ferring Pharmaceuticals, Saint-Prex, Switzerland), every other day, and hCG 5,000 IU (Choriomon^®^, Meizler, UCB, Biopharma, Belgium), every two weeks, both for three months.

The authors would like to apologize for any inconvenience caused.

